# Comparison of the chemical composition of major feed resources between the midland and lowland agro-ecological zones in the Majang zone of southwest Ethiopia

**DOI:** 10.1016/j.heliyon.2024.e35581

**Published:** 2024-07-31

**Authors:** Shimelis Assefa, Belay Duguma, Zemene Worku

**Affiliations:** Department of Animal Sciences, College of Agriculture and Veterinary Medicine, Jimma University, P.O. Box 307, Jimma, Oromia, Ethiopia

**Keywords:** Chemical composition, Crop residues, Improved forages, Natural pasture, Atella

## Abstract

Evaluation of feed chemical composition is essential for predicting nutrient deficiencies and improving feed for optimal livestock productivity. However, variation in agro-ecological zones (AEZs) might affect the chemical composition of feeds. The main objective of this study was to evaluate and compare the chemical composition of major feed resources in the midland and lowland AEZs of the Majang zone in southwest Ethiopia. A total of eight representative samples of feed resources were collected from lowland (Godere district) AEZ (500–1500 m above sea level) and midland (Mangashi district) AEZ (1501–2300 m above sea level). The samples were analysed for dry matter (DM), ash, crude protein (CP), ether extract (EE), neutral detergent fibre (NDF), acid detergent fibre (ADF) and acid detergent lignin (ADL). The result revealed significant differences (p < 0.05) in the chemical composition traits between the two AEZs in the study area. For example, the DM, ash and NDF contents of *C. dactylon* were higher (*P* < 0.05) in the lowland AEZ, while the ADF and ADL contents were higher in the midland; *D. abyssinica* had higher (*P* < 0.05) ADF content in the lowland, while *Z. mays* stover had higher (*P* < 0.05) DM, ash and ADF contents in the lowland. *S. bicolor* stover showed higher (*P* < 0.05) DM, ash, ADF and ADL contents in the lowland, while *C. gayana* had higher (*P* < 0.05) ash and CP contents in the lowland and higher (*P* < 0.05) ADF and ADL contents in the midland AEZ; Atella (a traditional alcohol byproduct) had greater (*P* < 0.05) ash and ADF contents in the lowland. Regardless of the AEZs, the DM, ash, NDF, ADF, EE, CP and ADL contents of the feed samples varied from 89.97 ± 0.33 % in *P. purpureum* to 92.50 ± 0.01 in Sorghum bicolor stover, 4.27 ± 0.19 in *Atella* to 12.37 ± 0.42 in elephant grass, 51.66 ± 0.38 in *Atella* to 66.10 ± 0.25 in *D. abyssinica*, 32.21 ± 0.29 in *D. abyssinica* to 42.58 ± 0.44 in *D. abyssinica*, 0.63 ± 0.15 in *Atella* to 2.55 ± 0.03 in *C. dactylon,* 4.77 ± 0.01 in Zea mays stover to 11.83 ± 0.02 in *V. unguiculata* and 10.31 ± 0.07 in Z. mays stover to 19.41 ± 0.07 % DM in *C. dactylon*, respectively. From this study it was concluded that variation in AEZ has a significant effect on chemical composition traits of the feeds in the study area. The results of the study revealed that *V. unguiculata* stands out as a valuable protein source, while crop residues may require additional protein supplementation to meet livestock requirements in the study area.

## 1 Introduction

Livestock production is an integral component of Ethiopian agriculture and contributes approximately 17–25.3 % of the total Gross Domestic Product (GDP), 39–45 % of the agricultural GDP, over 50 % of household income [1], 37–87 % of household income [2], and 60–70 % of the employment opportunities [3].

According to the Central Statistical Agency [4], Ethiopia has the largest livestock population in Africa estimated to be approximately 65.3 million cattle, 39.9 million sheep, 50.5 million goats, 2.1 million horses, 8.9 million donkeys, 0.4 million mules,7.7 million camels, and 59.5 million chickens. Despite Ethiopia's vast livestock resources, productivity has remained very low. This is attributed to various constraints including feed shortages, in both quality and quantity, high incidences of diseases and parasites, low genetic potential of indigenous livestock species, inadequate veterinary services, lack of access to credit, land scarcity, traditional husbandry practices and low adoption of improved technologies, among others [5,6]. According to Ref. [5], inadequate and low quality feed has been identified as the major constraint limiting livestock production in the traditional smallholder sector in Ethiopia.

According to a recent report by CSA [4], natural pastures, crop residues, hay, agro-industrial byproducts, improved forage and other products, such as animal byproducts, vegetable and fruit wastes are the main livestock feed resources in Ethiopia. However, most of these feed resources are low in both quality and quantity, which adversely affects livestock productivity [1].

Uchegbu et al. [7] have shown that feed is one of the most important aspects of livestock production accounting for 70 % of the cost of production. Onyagbodor and Oyedeji [8] have shown that the quality of feed resources is a major concern for farmers because it directly impacts on the overall quality of animal production. According to Owen et al. [9], year-round limited access to quality feed is the most common and universal challenge in SSA. Minson [10] also stated that the nutritional quality of feed is a major determinant of animal production in tropical areas. Forage quality is generally affected by environmental and management factors [11].

NDF content of feeds is inversely correlated with forage digestibility [12]. The lignin content of a plant is the most indigestible component of the fibre fractions and its amount will also influence the plant's digestibility [13]. It has been reported that the CP content of forages should be higher than the critical CP level of 7 % for optimal performance of ruminants [14], and the 8 % suggested by Ref. [15] for proper rumen function. Forages with CP content less than 8 % are categorized as poor/low quality forages resulting in less feed intake, lower digestibility and poor utilization of feed [16].

Teka et al. [17] have reported that the nutritional value of feed resources varies depending on environmental factors such as altitude, rainfall, soil type, cropping intensity, grazing land management and genetic characteristics specific to plant species. According to Schut et al. [18] accurately estimating the nutritional value of forages is crucial for livestock nutrition, as well as effectiveness of livestock production is linked to the availability of nutrients in the forage. Differences in forage species, soil nutrient levels, grazing pressure, management practices, temperature and agro-ecology are the main factors that influence the nutritive value of feedstuffs [17,[19], [20], [21], [22]].

Similar to other smallholder mixed crop livestock farming system in Ethiopia, natural pasture, crop residues, improved forages, and nonconventional feed resources such as *Atella* are the most commonly available feed resources for ruminants in the present study area. However, the chemical composition of these feed resources has never been documented under the different AEZs in the current study area in order to identify the limiting nutrients for targeted interventions. An evaluation of chemical composition of a feed is a valuable indicator of its nutritional quality, helpful in the formulation of balanced rations for better utilization and improvement of the commonly available feed resources and for predicting animal performance. Thus, to improve the quality of feed resources, it is necessary to obtain information of their chemical components in relation to animal requirements. Therefore, any study that contributes to better understating of feed quality in one of the lower-lying and relatively untapped areas of southwestern Ethiopia is very essential to generate information for further feed improvement and to enhance livestock productivity. The aim of the present study was to evaluate agroecological variations in the chemical composition of major livestock feed resources obtained from midland and lowland agro-ecological zones of southwest Ethiopia. The outcome of this study will aid in planning rational management and utilization of available feed resources, identifying nutrient deficiencies and suggesting supplemental requirements for livestock.

## 2 Materials and methods

### 2.1 Description of the study area

The study was conducted between November and January 2021 in two districts of Majang Zone, specifically; the Mangashi district (midland) and Godere district (lowland) AEZs of the Gambella National Regional State, southwest Ethiopia. The districts were purposely selected based on variations in the AEZ. The midland AEZ is located between 7°19′09″ latitude and 35°07′36″ longitude with an altitude ranging between 1500 and 2350 m above sea level. The mean annual minimum and maximum rainfall are 1500 and 2320 mm, respectively, with most of the rainfall occurring during the rainy season (from June to September). The mean annual minimum and maximum temperatures in the area are 27.5 and 32.5 °C, respectively. The lowland AEZ is situated at 7°25′52″ longitude and 35°06′16″ latitude with an elevation ranging between 500 and 1500 m above sea level. The mean annual minimum and maximum rainfall are 1000 and 1800 mm, respectively and most of the rainfall occurs during the rainy season (June to September). The mean annual minimum and maximum temperatures of the area are 28.5 and 34.5 °C, respectively. The farming system in the area is a mixed crop-livestock production system. Common crops cultivated include maize, sorghum, coffee, spices and root crops. Livestock species kept by farmers in the study area include cattle, goats, sheep, poultry, horses, mules and donkeys are the main livestock species kept by the farmers in the study area (Majang Zone Agricultural Development Office (MZADO, 2021).

### 2.2 Collection and preparation of feed samples

Eight representative feed samples used by livestock farmers in the studied area including *C. dactylon* (Bermuda grass)*,* Z. mays (maize) stover, *S. bicolor* (sorghum) stover, Atella (local brew byproduct), C. gayana (Rhodes grass), D. abyssinica (East African couchgrass), V. u*nguiculata* (cowpea) and *P. purpureum* (Elephant or Napier grass) were collected from the midland region. Additionally, six representative feed samples including *C. dactylon* (star grass, also known as Bermuda grass), Z. mays (maize) stover, *S. bicolor* (sorghum) stover, Atella (local brew byproduct), C. gayana (Rhodes grass), and D. abyssinica (East African couchgrass) were collected from the lowland AEZs between November and January 2021. The improved forages (*V. unguiculata* and *P. purpureum* were cultivated only by the midland farmers. The various feed samples were categorized into four types: natural pasture, crop residues, cultivated/improved forages and Atella. Maize and sorghum residues were collected from fields after the crops were harvested (with the grains already collected) and thoroughly dried in the field. Improved forages were obtained from plots of forages grown by farmers. Natural pasture species were collected from communal grazing lands, while *Atella* was obtained directly from households that supplemented it to their animals. Samples of natural pasture were collected using a 0.5m × 0.5m quadrate from each selected grazing site in each AEZ. The quadrate was thrown on the selected sites and sample collection of natural pasture and improved forages was accomplished by cutting aboveground biomass to mimic the way ruminants graze. After sampling, the same type of feed sample collected from each agro-ecological zone was bulked together, mixed thoroughly and sub-sampled to create one composite sample. The fresh samples of natural pasture, improved forages and *Atella* were weighed, sun-dried and stored in polythene bags before being transported to the Animal Nutrition Laboratory of the College of Agriculture and Veterinary Medicine at Jimma University.

### 2.3 Chemical analysis

In the laboratory, the samples were oven dried at 60 °C overnight to determine the chemical composition of feed resources. The dried samples were then ground to pass through a 1 mm screen using a Wiley sieve. The samples were analysed on a % DM basis for proximate analysis to determine ash, EE and CP following the methods of the Association of Official Analytical Chemists [23]. The DM content was determined by drying the samples at 105 °C overnight. Total ash content was determined by oven drying the samples at 105 °C overnight and combusting the samples in a muffle furnace at 550 °C for 8 h. Nitrogen (N) content was determined by the micro-Kjeldahl digestion, distillation and titration method and the CP content was estimated by multiplying the N content by 6.25. The structural components (NDF, ADF and ADL) were determined according to Van Soest et al. [24]. In the laboratory, duplicate samples of each feed type were used to determine the chemical composition.

### 2.4 Statistical analysis

All the data were analysed using the Statistical Package for the Social Sciences (SPSS) software version 20. Descriptive statistics such as the mean, and standard error (SE) were generated. Additionally, an independent sample *t*-test was used to compare differences in the means of chemical composition variables between the midland and lowland agro-ecological zones. Differences were deemed significant at the p < 0.05 level.

### 2.5 Limitations of the study

The main limitations of the study were the lack of laboratory facilities for evaluating the in vitro dry matter digestibility (IVDOMD) of feed samples and financial constraints in evaluating the chemical composition of indigenous fodder trees and shrubs available in the study area.

## 3 Results and discussion

### 3.1 Chemical composition of the natural pasture

Table 1 shows the chemical composition (on a % DM basis) of natural pasture grass species. The major natural pasture species found in the study area are include *C. dactylon* and *D. abyssinica.* The results indicated that the two AEZs showed significant differences for certain chemical composition traits of natural pasture species. The *C. dactylon* had significantly (p < 0.05) higher DM, ash, NDF, ADF and ADL contents in the lowland as compared to in the midland AEZ. The differences might be attributed to the variations between the two AEZs in climate, soil rainfall and temperature. *D. abyssinica* had significantly (*P* < 0.05) higher ADF in the lowland than in the midland AEZ. *C. dactylon* had higher CP, ash, EE and ADL content than did *D. abyssinica*, while *D. abyssinica* had higher DM, NDF and ADF content than did C. *dactylon.* This difference could be attributed to variation in species, maturity stage and management practices.

**Table 1:** Chemical composition (% DM) of natural pastures collected from Mangashi (midland) and Godere (lowland) districts of Majang zone in southwest Ethiopia.

	Agro-ecological zone					
	Midland	Lowland	*p*-value	Midland	Lowland	*p*-value
Feed type	*C. dactylon*			*D. abyssinica*		
Chemical composition (Mean ± SE)						
DM	89.48 ± 0.01^b^	89.65 ± 0.02^a^	0.028	91.10 ± 0.42	90.22 ± 0.92	0.475
Ash	9.36 ± 0.02^b^	9.94 ± 0.03^a^	0.004	8.60 ± 0.52	8.62 ± 0.59	0.982
NDF	63.82 ± 0.06^b^	65.68 ± 0.06^a^	0.002	66.10 ± 0.25	64.67 ± 2.83	0.665
ADF	39.72 ± 0.04^b^	34.85 ± 0.04^a^	0.000	32.21 ± 0.29^b^	42.58 ± 0.44^a^	0.007
EE	2.55 ± 0.03	2.34 ± 0.03	0.211	1.69 ± 0.27	1.57 ± 0.22	0.775
CP	11.22 ± 0.04^b^	11.45 ± 0.31^a^	0.536	9.95 ± 0.16	9.79 ± 0.75	0.850
ADL	19.41 ± 0.07^a^	16.32 ± 0.05^b^	0.001	16.77 ± 0.70	19.09 ± 0.55	0.121

Means in a row with different superscripts are significantly different (*P* < 0.05). ADF, acid detergent fibre; ADL, acid detergent lignin; CP, crude protein; DM, dry matter; EE, ether extract; NDF, neutral detergent fibre.

The average DM content of natural pasture varied from 89.48 ± 0.01 % in *C*. *dactylon* in the midland to 91.10 ± 0.42 % in *D. abyssinica* in the midland AEZ. This variation might be due to differences in species, agro-ecology, soil and stage of maturity. The DM values of natural pasture reported in this study are greater than the recommended range of 70–80 % [25], which may limit feed intake. However, our results are comparable to the findings of [26], who reported DM values of 92.26 ± 0.55 % for *C. dactylon* and 91.40 ± 0.84 % for *D. abyssinica.* The DM content of *C. dactylon* recorded in this study is slightly lower than the findings of Urge et al. [27], who reported 90.75 %. However, the DM value of natural pasture in this study did not concur with the findings of [28], who reported values varying from 18.8 % to 75.5 %. The variations in DM values between the results of this study and those of different literature reports could be due to differences in forage species or variety, stage of maturity at sampling, climate and soil fertility. DM levels of fodder and formulated feeds affect the availability of nutrients and their microbial activities (McDonald et al. [29]. For instance, fodders and forages with a DM content of 85 % become easily breakable and lose their readily digestible fractions. Generally, the high DM content of natural pasture species recorded in this study could be due to the time of sampling (the dry season), maturity stage and a decline in rainfall.

The mean ash content of natural pasture species varied from 8.60 ± 0.52 % in *D. abyssinica* in the midland to 9.94 ± 0.03 % in *C. dactylon* in the lowland AEZ. This variation could be due to differences in species, soil fertility level, climate and stage of plant maturity. The ash content of natural pasture species reported in this study is lower than the findings of earlier work [26], who reported 14.85 ± 0.44 in *C. dactylon* and 9.25 ± 1.12 % DM in *D. abyssinica*. Gurmessa et al. [30] also reported ash content of natural pasture ranging between 9.21 % and 16.9 %.

The average NDF content of natural pasture varied from 63.82 ± 0.06 % in *C. dactylon* in the midland to 66.10 ± 0.25 % DM in *D. abyssinica* in the midland AEZ*.* The present NDF values are lower than the 70.14 ± 1.98 in *C. dactylon* and 64.67 ± 1.51 % DM in *D. abyssinica,* as reported by Ref. [26] and 76 % [31]. However, our findings exceeded the threshold level (60 % DM), indicating lower voluntary intake. According to Van Soest [32], feedstuffs with NDF content of above 60 % are classified as poor and from 50 to 60 % as moderate quality. The same author [25] stated that an NDF content of feeds above 55 % limits DM intake and eventually animal productivity. It has been reported that a high fibre content of forages suggests lower cell content and digestibility, and this implies the need to incorporate other forage supplements, such as browse fodder, into basal diets [24].

The mean ADF content of natural pasture in the present study varied from 32.21 ± 0.29 % in *D. abyssinica* in the midland to 42.58 ± 0.44 % DM in *D. abyssinica* in the lowland AEZ. The current ADF values are lower than the 49.37 ± 1.22 % in *C. dactylon* and 50.51 ± 1.30 % DM in *D. abyssinica* [26]. A high ADF content of forages is due to increased lignification of cellulose in plants, resulting in reduced digestibility and limited intake of other feeds [33,34].

The mean EE or crude fat content of natural pasture varied from 1.57 ± 0.22 in *D. abyssinica* in the lowlands to 2.55 ± 0.03 % DM in *C. dactylon* in the lowlands AEZ. These values are higher than 1.31 ± 0.11 in *C. dactylon* but slightly lower than 1.58 ± 0.11 % DM in *D. abyssinica* [26]. The EE content of *C. dactylon* and *D. abyssinica* obtained in the current study are lower than the minimum level (7 % DM)-above this limit may increase methane production and energy inefficiency in ruminants, and limit feed and fodder intake [35]. An EE composed of unsaturated fatty acids in feeds can affect ruminal flora than those containing up to 5–6% DM [36].

The mean CP content of natural pasture in this study varied from 9.79 ± 0.75 % in *D. abyssinica* in the lowland to 11.45 ± 0.31 % DM in *C. dactylon* in the lowland AEZ. These values are comparable with the findings of [26], who reported values of 12.07 ± 0.44 in *C. dactylon* and 9.54 ± 0.46 % DM in *D. abyssinica*. The CP value of *C. dactylon* recorded in current study is lower than the findings of [33], who observed 13.39 %. It has been reported that ruminant livestock require feedstuffs with minimum CP content of 11 % for maintenance and 13 % for growth [37]. Earlier studies revealed that the CP content of tropical forages is highly variable ranging from as low as 4 % [38] to as high as 36 % [Martens et al., 2012]. On the contrary, Paul et al. [39] reported that the CP content of tropical grass species ranged from as low as 4 % to as high as 15.1 %. In general, the CP concentrations of natural pasture in the current findings is higher than the minimum level of 7–8 % required for optimal rumen functioning, and microbial growth and activity in the rumen [40,41].

The mean ADL content of natural pasture in this study varied between 16.32 ± 0.05 % in *C. dactylon* in the lowland and 19.41 ± 0.07 % in *C. dactylon* in the midland AEZ. These current values are higher than the maximum recommended level of 7 % and could limit DM intake [42]. Van Soest [32] had reported lignin content of feeds above 6.0 % DM affects digestibility of forages negatively. Mean ADL content of *C. dactylon* in this study is higher than the findings of [27] who observed 9.3 % and [43] who reported 2.53 %. Climate, soil type and fertility, stage of maturity stage, forage species or variety and management practices are the factors which affect the chemical composition of feeds [44].

### 3.2 Chemical composition of crop residues

The chemical compositions of *Z. mays* and *S. bicolor* stovers in the study area are shown in Table 2. Maize and sorghum stovers were the main sources of dry season roughage for feeding livestock in the current study area. The results revealed that certain chemical composition traits *Z. mays* and *S. bicolor* stovers varied significantly (*P* < 0.05) between the AEZs. contents of the *Z. mays* stover had significantly (*P* < 0.05) higher DM, ash and ADF in lowland AEZ, while its ADL content was significantly (*P* < 0.05) higher in midland AEZ. The variation could be attributed to differences in climate, soil and temperature. Mean content of NDF, EE and CP contents of *Z. mays* did not differ significantly (*P* > 0.05) between the two AEZs. *S. bicolor* stover significantly (p < 0.05) higher DM, ash, ADF and ADL in lowland AEZ, whereas its NDF content significantly (*P* < 0.05) higher in midland AEZ. Mean EE and CP contents of *S. bicolor* stover did not differ significantly (*P* > 0.05) between the two AEZs. *Z. mays* had significantly (*P* < 0.05) higher NDF and EE than *S. bicolor* stover, while ash, EE, CP and ADL contents were significantly (*P* < 0.05) higher in *S. bicolor* stover than in *Z. mays* stover*.* These differences might be attributed to variation in crop species.

**Table 2:** Chemical composition (% DM) of maize and sorghum stovers collected from the Mangashi (midland) and Godere (lowland) districts of southwest Ethiopia.

Feedstuff	*Z. mays*			*S. bicolor*		
AEZ	Midland	Lowland	*p*-value	Midland	Lowland	*p*-value
Chemical composition (Mean ± SE)						
DM	91.16 ± 0.02^b^	91.44 ± 0.04^a^	0.028	90.93 ± 0.03^b^	92.50 ± 0.01^a^	0.000
Ash	5.85 ± 0.02^b^	7.83 ± 0.02^a^	0.000	7.19 ± 0.01^b^	8.33 ± 0.03^a^	0.001
NDF	64.24 ± 0.02	64.34 ± 0.03	0.121	64.76 ± 0.05^a^	61.44 ± 0.04^b^	0.000
ADF	38.52 ± 0.02^b^	40.06 ± 0.02^a^	0.000	37.63 ± 0.05^b^	39.35 ± 0.04^a^	0.002
EE	0.68 ± 0.01	0.66 ± 0.02	0.633	0.84 ± 0.01	0.82 ± 0.02	0.564
CP	4.77 ± 0.01	4.82 ± 0.02	0.241	7.40 ± 0.05	7.48 ± 0.01	0.312
ADL	12.28 ± 0.07^a^	10.31 ± 0.07^b^	0.003	18.68 ± 0.05^b^	19.46 ± 0.14^a^	0.037

Means in a row with different superscripts are significantly different (*P* < 0.05). ADF, acid detergent fibre; ADL, acid detergent lignin; CP, crude protein; DM, dry matter; EE, ether extract; NDF, neutral detergent fibre.

The average DM content of crop stovers in this study varied from 90.93 ± 0.03 in *S. bicolor* in the lowland to 92.50 ± 0.0 % DM in *S. bicolor* in the lowland AEZ. These values concur with the findings of [26], who reported DM contents of 92.55 ± 0.70 % in *Z. mays* and 93.63 ± 0.29 % in *S. bicolor* stovers. The DM content of crop stovers recorded in this study is higher than the recommended level of 70–80 %, which may limit feed intake [25]. The high DM content of the crop stovers observed in this study could be attributed the collection of samples after the crops had been thoroughly dried. At this DM level, the stovers can be stored for longer periods since they may inhibit microbial growth and mould development.

The mean ash content of crop stovers varied from 5.85 ± 0.02 % in *Z. mays* in midland to 8.33 ± 0.03 % DM in *S. bicolor* in lowland AEZ. These values are lower than the results of [26], who reported 8.12 ± 0.57 % in *Z. mays* stover and 7.20 ± 0.30 % ash in *S. bicolor*.

The mean CP content of crop stovers ranged between 4.77 ± 0.01 % in *Z. mays* in midland and 7.48 ± 0.01 in *S. bicolor* in lowland AEZ. These results are not consistent with the findings of [26], who reported 3.78 ± 0.10 % in *Z. mays* stover and 3.53 ± 0.17 % in *S. bicolor* stover. The CP content of crop stovers observed in this study are lower than the minimum level of 7–8 % CP required for microbial growth and activity in the rumen [16,25]. CP content in feeds less than 7 % adversely affects rumen microbial activity [32]. In another report, Asaolu et al. [45] stated that ruminant livestock require feed resources with recommended CP contents of 11 % for maintenance and 13 % for growth. Thus, the CP contents of crop stovers obtained in this study cannot support adequate microbial growth or the recommended animal's requirements unless supplemented with protein source of feeds or treated with urea [9].

The mean NDF content of crop stovers varied from 61.44 ± 0.04 % in *S. bicolor* stover in the lowland to 64.76 ± 0.05 % in *Z. mays* stover in the midland AEZ. The values are lower than the results of [26], who observed 78.19 ± 0.77 % in *Z. mays* stover and 74.44 ± 2.07 % in *S. bicolor* stover. It was observed that *Z. mays* and *S. bicolor* stovers had the highest NDF content as compared to the other feed resources evaluated in this study, indicating limited intake and digestibility. Fondevila et al. [46] had reported that the low nutritive value and digestibility of cereal straws limits their use as animal feed. To overcome this problem, different crop residues treatment methods have been developed to increase their nutritive value and digestibility through structural modifications of the polysaccharide–lignin crosslinks of the cell wall [46,47]. Among these methods, alkali and urea treatments as a source of ammonia has been documented [9]. The NDF values of crop stovers observed in this study are lower than the findings of [38], who reported 46.79–56.63 % NDF in crop stovers. Earlier works reported that NDF content of roughage feeds below 45 %, 45–65 % and above 65 % are classified as high, medium and low quality feeds, respectively [48]. Based on this category, the maize and sorghum stovers in this study can be categorized as medium quality roughages as their NDF content ranged from 61.44 % to 64.76 %.

The average ADF content of crop stovers varied from 37.63 ± 0.05 % in *S. bicolor* in the midland to 40.06 ± 0.02 % DM in *Z. mays* stover in the lowland AEZ. The values are higher than the results of [26], who reported 55.37 ± 2.12 % in *Z. mays* stover and 58.70 ± 0.82 % in *S. bicolor* stovers. However, the values of ADF obtained in this study are lower than the findings of [38] who observed values ranging from 68.48 to 70.63 % DM.

It was found that the mean EE content of crop stovers varied from 0.66 ± 0.02 % in *Z. mays* stover in the lowland to 0.84 ± 0.01 % in *S. bicolor* in the midland AEZ. The values are lower than the results of [26], who observed 1.79 ± 0.12 % in *Z. mays* stover and 1.40 ± 0.12 % in *S. bicolor*. According to Ref. [35], an EE content of feeds higher than 7 % limits l feed intake.

The mean ADL content of crop stovers varied from 10.31 ± 0.07 % in *Z. mays* stover in the lowland to 19.46 ± 0.14 % DM in *S. bicolor* in the lowland AEZ. Mulushewa et al. [49] reported higher ADL content of *Z. mays* (11.76 % DM) and lower ADL content of *S. bicolor* stovers (12.76 % DM) than the current findings. Generally, the variations observed in chemical composition traits between the two crop stovers evaluated in this study might be due to the crop species, stage of maturity, soil fertility status, agronomic practices and agro-ecology.

### 3.3 Chemical composition of improved forages

The chemical composition of cultivated/improved forages is shown in Table 3. The results revealed that the chemical composition of cultivated forages varied significantly (p < 0.05) between the two agro-ecological zones of the studied area. Relatively higher values of ash and CP content was found in *C. gayana* collected from lowland than did those from midland AEZ (*P* < 0.05). On the other hand, *C. gayana* collected from midland had significantly (p < 0.05) higher ADF and ADL contents. However, DM, EE and NDF content of *C. Gayana* at the two AEZs was not-significant (*P* > 0.05). The results revealed a significant difference (*P* < 0.05) in chemical composition traits between the species of cultivated forages evaluated in this study. It was observed that the species varied in their chemical composition. For example, *C. gayana* had higher DM, NDF, and EE values than *V. unguiculata,* whereas ash, CP and ADL values were higher in *V. unguiculata* than *C. gayana.* Additionally, *P. purpureum* had a higher ADF content than both *C. gayana* and *V. unguiculata.* The lowest CP value was observed in *C. gayana* as compared to the other species. the variations observed in the chemical composition traits of improved forages in this study could be attributed to differences in forage species, altitude, climate and soil factors.

**Table 3:** Chemical composition (% DM) of cultivated forages collected from Mangashi (midland) and Godere (lowland) districts of southwest Ethiopia.

Feedstuff	*C. gayana*		*p*-value	*V. unguiculata*	*P. purpureum*
AEZ	Midland	Lowland		Midland	Lowland
Chemical composition (Mean ± SE)					
DM	90.77 ± 0.02	90.66 ± 0.03	0.099	90.38 ± 0.00	89.97 ± 0.33
Ash	9.52 ± 0.02^b^	10.94 ± 0.03^a^	0.001	11.37 ± 0.10	12.37 ± 0.42
NDF	60.02 ± 0.01	60.03 ± 0.03	0.817	53.35 ± 1.03	52.19 ± 0.32
ADF	35.76 ± 0.06^a^	32.44 ± 0.03^b^	0.000	40.80 ± 0.04	41.74 ± 0.04
EE	1.54 ± 0.02	1.64 ± 0.04	0.115	1.17 ± 0.03	1.60 ± 0.08
CP	9.18 ± 0.03^b^	9.42 ± 0.03^a^	0.034	11.83 ± 0.02	10.20 ± 0.02
ADL	16.60 ± 0.10^a^	15.41 ± 0.03^b^	0.008	19.06 ± 0.01	18.66 ± 0.08

The mean DM content of cultivated forage species in this study ranged from 89.97 ± 0.33 % in *P. purpureum* in the midland to 90.77 ± 0.02 % in *C. gayana* in the midland AEZ. The high DM values of the cultivated forages evaluated in this work indicate their advanced age and the high temperature during the sampling period (dry season). The DM values of cultivated forages in this study are above the critical values of 70 %–80 % as recommended by Ref. [25]. The DM contents of *C. gayana* and *P. purpureum* recorded in this study are lower than the results of [49], who reported DM levels of 93.3 % in *C. gayana* and 92.7 % in *P. purpureum.*

The mean ash content of improved forages varied from 9.52 ± 0.02 % in *C. gayana* in the midland to 12.37 ± 0.42 in *P. purpureum* in the lowland. The variations may be due to agro-ecology, forage species, maturity stages, and soil fertility differences. The values observed in this study are higher than the findings of [49] who reported ash values of 6.33 % in *C. gayana* and 8.2 % DM in *P. purpureum*, The mean NDF content of improved forages ranged from 52.19 ± 0.32 in *P. purpureum* in lowland to 60.03 ± 0.03 in *C. gayana* in midland. The NDF values are lower when compared to the results of [49], who reported 65.8 % for *C. gayana* and 70.8 % for *P. purpureum*. The variations could be attributed to climate, soil status, maturity stage and management practices. According to the classification of roughages based on NDF content, the improved forages evaluated in this study can be classified as medium quality feeds [48].

The mean ADF content of cultivated forages in the present study varied from 32.44 ± 0.03 % in *C. gayana* to 41.74 ± 0.04 in *P. purpureum*. The ADF values are lower than earlier study by Ref. [49], who found 52.9 % in *C. gayana* and 54.9 % in *P. purpureum*. The variations may be attributed to climate, soil status, plant variety, stage of maturity and management practices. Van Saun [50] reported that feedstuffs with greater than 35 % ADF content are considered low-quality feeds, and ADF content and digestibility of feeds are negatively correlated [40]. The mean EE content of improved forages in the present work varied from 1.17 ± 0.03 in *V. unguiculata* in the midland to 1.64 ± 0.04 in the *C. gayana* in the lowland AEZ.

The average CP content of improved forages varied from 9.18 ± 0.03 in *C. gayana* collected from the midland to 11.83 ± 0.02 in *V. unguiculata* from the midland. The observed variations may be due to forage species, soil factors, climate, and agro-ecological zone. The CP content of all cultivated forages in this study was above the minimum (7.0–8%) level required for ideal ruminal fermentation [25]. In this study, V*. unguiculata* had the highest CP content compared to the other improved forages, indicating its potential as a supplement to low quality crop residues and natural pasture especially in the dry season.

The mean ADL content of improved forages ranged from 15.41 ± 0.03 in *C. gayana* in the lowland and 19.06 ± 0.01 % DM in *V. unguiculata* in the midland AEZ. The ADL values are higher when compared with the minimum recommended (7 % DM) level. This may ultimately affect feed intake and digestibility negatively. In general, the variations that were observed in the chemical composition traits among the evaluated improved forages could be due to agro ecological zone, species, stage of maturity, climate, soil fertility status and agronomic practices.

### 3.4 Chemical composition of atella

Table 4 presents the chemical composition of *Atella* in the study area*.* The DM content of *Atella* ranged from 91.51 ± 0.00 at midland to 91.64 ± 0.04 % DM at lowland AEZs. The DM values of Atella in the current study are comparable with the findings of [26], who reported 91.72 ± 0.95 %. The mean ash content of *Atella* varied from 4.27 ± 0.19 in the midland to 7.53 ± 0.06 % DM in the lowland AEZ and differed significantly (P < 0.05) between the two AEZs. Earlier studies [26] have reported higher ash content (7.92 ± 0.19 %) of *Atella* than our findings.

**Table 4:** Chemical composition (% DM) of *Atella* collected from Mangashi (midland) and Godere (lowland) districts of Majang zone, southwest Ethiopia.

Feedstuff	*Atella*		*p*-value
AEZ	Midland	Lowland	
Chemical composition (Mean ± SE)			
DM	91.51 ± 0.00	91.64 ± 0.04	0.090
Ash	4.27 ± 0.19^b^	7.53 ± 0.06^a^	0.004
NDF	51.66 ± 0.38	52.47 ± 0.03	0.168
ADF	34.20 ± 0.03^b^	38.27 ± 0.15^a^	0.000
EE	0.72 ± 0.15	0.63 ± 0.15	0.051
CP	5.11 ± 0.03	5.30 ± 0.04	0.063
ADL	18.36 ± 0.46	18.51 ± 0.26	0.811

Means in a row with different superscripts are significantly different (*P* < 0.05). ADF, acid detergent fibre; ADL, acid detergent lignin; CP, crude protein; DM, dry matter; EE, ether extract; NDF, neutral detergent fibre.

The mean NDF content of the *Atella* ranged from 51.66 ± 0.38 in the midland to 52.47 ± 0.03 % DM in the lowland region. The variation may be due to the grain type and methods used for the preparation of *Tella* (tradition alcohol drink). The NDF content of *Atella* observed in this study is lower than the findings of [51] who found 54.0 %. The mean ADF content of *Atella* ranged from 34.20 ± 0.03 in the midland and 38.27 ± 0.15 % DM in the lowland. The ADF values obtained in current study are higher than the results of [51], who reported 29.0 %.

The mean EE content of *Atella* varied from 0.63 ± 0.15 in the lowland to 0.72 ± 0.15 % DM in the midland. These values are lower than the findings of [26], who reported 1.56 ± 0.09 %. Feeds with low EE content imply low energy content and such feeds should be supplemented with high energy feeds [52].

The mean CP content of *Atella* obtained in this study varied from 5.11 ± 0.03 in the midland to 5.30 ± 0.04 % DM in the lowland. The values are very much lower compared the findings of [53], who reported 17.6 % CP in *Atella*. It is also lower than the minimum recommended (7 %) level required for rumen microbial protein synthesis and maintenance requirement of ruminants [32]. According to Ref. [54] feeds that contain <12 %, 12–20 %, and >20 % CP are categorized as low, medium, and high protein sources, respectively. Therefore, the CP content of *Atella* found in the present study can be classified as low, which is unlikely to provide the minimum CP level required for rumen microbial growth.

The ADL content of *Atella* obtained in this study ranged from 18.36 ± 0.46 17.6 % in midland to 18.51 ± 0.26 % DM in the lowland, with no significant difference between the two AEZs. The values are greater than the minimum (7 %) and could limit digestibility. According to Van Soest [32] ADL content of feeds above 6 % affects the overall digestibility and limits nutrient availability [25]. It has been reported that lignin is the single most important factor limiting feed intake, rate of organic matter fermentation, number of microbial cells produced per unit of fermented organic matter, and the proportion of propionate to acetate in the products of fermentation [55]. Generally, the observed variation in chemical compositions of *Atella* between the current findings and reported literature earlier could be due to crop type and methods used for the preparation of *Tella*, a traditional alcoholic drink in Ethiopia. Tella is an Ethiopian traditional fermented beer-like beverage made from a variety of cereals and gesho (Rhamnus prinoides) instead of hops. Tella is similar to beer in that it is made from malted barley and other grains, with the addition of gesho as a traditional substitute for hops [56].

## 4 Conclusions

From this study it was concluded that variation in AEZ had a significant effect on chemical composition traits of the major feed resources in the study area. This might be attributed to variations in soil factors, climatic condition, rainfall and temperature. Therefore, this study would bring attention of researchers for new feed management approaches and utilization practices along the different agroecological zones. The CP content of natural pasture and cultivated forages is sufficient for a medium level of livestock production. The results of the study revealed that *V. unguiculata* stands out as a valuable protein source, while crop residues may require additional protein supplementation to meet livestock requirements in the study area. Based on the findings of this study, it is recommended that variations in agro-ecological zones should be considered when evaluating the chemical composition of feeds to provide accurate nutritional information. Moreover, further animal feeding trial study is needed to substantiate the current findings.

## Data availability statement

Data will be available upon request.

## Ethics statement

Informed consent was not required for this study because deeply personal or confidential.

## CRediT authorship contribution statement

**Shimelis Assefa:** Writing – original draft, Methodology, Investigation, Formal analysis, Data curation, Conceptualization. **Belay Duguma:** Writing – review & editing, Validation, Supervision, Formal analysis, Data curation, Conceptualization. **Zemene Worku:** Writing – review & editing, Validation, Supervision, Methodology, Investigation, Data curation, Conceptualization.

## Declaration of competing interest

The authors declare that they have no known competing financial interests or personal relationships that could have appeared to influence the work reported in this paper.

## References

[1] Shapiro B.I., Gebru G., Desta S., Negassa A., Nigussie K., Aboset G., Mechale H. (2017).

[2] GebreMariam S., Amare S., Baker D., Solomon A., Davies R. (2013).

[3] Azage T., Berhanu G., Hoekstra D., Berhanu B., Yoseph M. (2013).

[4] CSA (Central Statistical Agency) (2020). Agricultural Sample Survey 2019/20 [2012 E.C.]: Volume II. Report On Livestock And Livestock Characteristics (Private Peasant Holdings) Ethiopia; Statistical Bulletin 587.

[5] Duguma B., Janssens G.P.J. (2021). Assessment of livestock feed resources and coping strategies with dry season feed scarcity in mixed crop–livestock farming systems around the gilgel gibe catchment, southwest Ethiopia. Sustainability.

[6] Welay G.M., Tedla D.G., Teklu G.G., Weldearegay S.K., Shibeshi M.B., Kidane H.H., Gebrezgiabher B.B., Abraha T.H. (2018). A preliminary survey of major diseases of ruminants and management practices in Western Tigray province; northern Ethiopia. BMC Vet. Res..

[7] Uchegbu M.C., Irechukwu N.M., Omede A.A., Nwaodu C.H., Anyanwu G.A., Okoli I.C., Udedibie A.B.I. (2009).

[8] Onyagbodor O.P., Oyedeji J.O. (2021). Quality assessment of some commercially produced animal feeds and two native forages in southern Nigeria. Zool..

[9] Owen E., Jayasuriya M.C.N. (1989). Use of crop residues as animal feeds in developing countries. Res. Dev. Agric..

[10] Minson D.J. (1990).

[11] Getnet A., Ledin I. (2001). Effect of variety, soil type and fertilizer on the establishment, growth, forage yield and voluntary intake by cattle of oats and vetches cultivated in pure and stands and mixtures. Anim. Feed Sci. Technol..

[12] McDonald P., Edwards R.A., Green J.F.D., Halgh A.M.C. (1995).

[13] Aremu O.T., Onadeko S.A., Inah E.I. (2007). Impact of grazing on forage quality and quantity for ungulates of the Kainji Lake National Park, Nigeria. J. Appl. Sci..

[14] NRC (1981).

[15] Norton B.W., Gutteridge R.C., Shelton H.M. (1994). Forage Tree Legumes in Tropical Agriculture.

[16] Leng R.A. (1990). Factors affecting the utilization of poor quality forage by ruminants particularly under tropical condition. Nutr. Res. Rev..

[17] Teka H., Madakadze I.C., Angassa A., Hassen A. (2012). Effect of seasonal variation on the nutritional quality of key herbaceous species in semi-arid areas of Borana, Ethiopia. Indian J. Anim. Nutr..

[18] Schut A.G.T., Gherardi S.G., Wood D.A. (2010). Empirical models to quantify the nutritive characteristics of annual pastures in south-west Western Australia. Crop Pasture Sci..

[19] Teklu B., Negesse T., Angassa A. (2010). Effects of farming systems on floristic composition, yield and nutrient content of forages at the natural pasture of Assosa zone (western Ethiopia). Tropical and Subtropical Agroecosystems.

[20] Kelkay Z.T., de Boer W.F., Baars R.M.T., Prins H.H.T. (2011). Changes in soil nutrients, vegetation structure and herbaceous biomass in response to grazing in a semi-arid savanna of Ethiopia. J. Arid Environ..

[21] Henkin N.E.D., Ungar L., Davsh A., Perevolotsky Y., Yehuda M., Sternbergs H., Voet S.Y.L. (2011). Effects of cattle grazing on herbage quality in herbaceous Mediterranean rangeland. Grass Forage Sci..

[22] Mengistu A., Aster A. Ayana A. (2015). Herbaceous Biomass and Nutritional Quality in Southeast Ethiopia.

[23] AOAC (2005) Association of Official Analytical Chemist, Official Methods of Analysis. 18th Edition, AOAC International, Suite 500, 481 North Frederick Avenue, Gaithersburg, Maryland 20877-2417, USA. AOAC (Association of Official Analytical Chemists), Off. Methods Anal. AOAC Int. (OMA) (2005).

[24] Van Soest P.J., Robertson J.B., Lewis B.A. (1991). Methods for dietary fibre, neutral detergent fibre and nonstarch polysaccharides in relation to animal nutrition. J. Dairy Sci..

[25] Van Soest P.J. (1994).

[26] Bayissa T., Duguma B., Desalegn K. (2022). Chemical composition of major livestock feed resources in the medium and low agroecological zones in the mixed farming system of Haru District, Ethiopia. Heliyon.

[27] Urge M., Hundie D., Gobena G. (2018).

[28] Chalchissa G., Mekasha Y., Urge M. (2014). Feed resources quality and feeding practices in urban and peri-urban dairy production of southern Ethiopia. Tropical and subtropical Agroecosystems.

[29] McDonald P., Edwards R.A., Greenhalgh J.F.D., Morgan C.A., Sinclair L.A., Wilkinson R.G. (2011).

[30] Gurmessa K., Tolemariam T., Tolera A., Beyene F., Demeke S. (2015). Feed resources and livestock production situation in the highland and mid altitude areas of Horro and Guduru Districts of Oromia Regional State, Western Ethiopia. Sci. Technol. Arts Res. J..

[31] Diriba G., Hailemariam M., Ashenafi M., Adugna T. (2012). Herbage yield, species diversity and quality of native grazing land vegetation under sub humid climatic conditions of Western Ethiopia. J. Agric. Res. Develop.

[32] Van Soest P.J. (1982).

[33] Gelaye K.T. (2015).

[34] Depeters E. (1993). California Alfalfa Workshop (Eds) Proceedings of the 23rd California Alfalfa Symposium at Fresno, California, USA.

[35] Eastridge M.L. (2014). Moderation, to Dairy Cows.

[36] Ramos L., Apráez J., Cortes K., Apráez J. (2021). Nutritional, antinutritional and phenological characterization of promising forage species for animal feeding in a cold tropical zone. Revista de Ciencias Agrícolas.

[37] Asaolu V., Binuomote R., Akinlade J., Oyelami O., Kolapo K. (2011). Utilization of Moringa oleifera fodder combinations with *Leucaena leucocephala* and *Gliricidia sepium* fodders by West African dwarf goats. Int. J. Agric. Res..

[38] Akinfemi A., Adesanya A.O., Aya V. (2009). Use of an in vitro gas production technique to evaluate some Nigerian feedstuffs. Am.-Eurasian J. Sci. Res..

[39] Paul S., Sebastian M., Appolinaria P., Eliot C.P., Sakurani B., Jelantik I., Torben H., Martin R.W., Jørgen M. (2006). Feed value of selected tropical grasses, legumes and concentrates. Vet. Arh..

[40] McDonald P., Edwards R.A., Greenhalgh J.F.D., Morgan C.A. (2002).

[41] Detmann C.B. Sampaio E., Lazzarini I., de M.A., Paulino Souza M.F., de Campos S., Filho V. (2009). Rumen dynamics of neutral detergent fibre in cattle fed low-quality tropical forage and supplemented with nitrogenous compounds. Rev. Bras. Zootec..

[42] Reed J.D., Yilma K., Fossel L.K. (1988). Proceedings of a Workshop Held at ILCA, Addis Ababa, Ethiopia, 7-10 December 1987. Addis Ababa.

[43] Andualem T., Berhan T., Gebeyehu G., Ermias B. (2015). Characterization of cattle husbandry practices in Essera Woreda; Dawuro zone; southern Ethiopia. Afr. J. Agric. Res..

[44] Rojas-Downing M.M., Pouyan N.A., Harrigan T., Woznicki S.A. (2017). Climate change and livestock: impacts, adaptation, and mitigation. Climate Risk Management.

[45] Asaolu V., Binuomote R., Akinlade J., Oyelami O., Kolapo K. (2011). Utilization of Moringa oleifera fodder combinations with *Leucaena leucocephala* and *Gliricidia sepium* fodders by West African dwarf goats. Int. J. Agric. Res..

[46] Munoz M. Fondevila F., Joy M., Castrillo C. (1990). Utilizaci ~ on de la paja de cereales: tratamientos y suplementacion. Ovis.

[47] Flachowsky G., Kamra D.N., Zadrazil F. (1999). Cereal straws as animal feed—possibilities and limitations. J. Appl. Anim. Res..

[48] Singh G.P., Oosting S.J. (1992). A model for describing the energy value of straws. Indian dairy man XLIV.

[49] Mulushewa S., Diba D., Duressa D. (2018). Availability and nutritional potential of major feed resources at wayutuka district, East Wollega, Ethiopia. Acad. J. Plant Sci..

[50] Van Saun R.J. (2006). Determining forage quality: understanding feed analysis. Lamalink. Com.

[51] Solomon D. (2007). Comparative nutritive value of *Atella* and industrial brewers grains in chicken starter ration in Ethiopia. Livest. Res. Rural Dev..

[52] Babayemi O.J., Bamikole M.A. (2006). Effects of Tephrosia candida DC leaf and its mixtures with Guinea grass on in vitro fermentation changes as feeds for ruminants in Nigeria. Pak. J. Nutr..

[53] Kassahun G., Taye T., Adugna T., Fekadu B., Solomon D. (2012). Productive and reproductive performance of horro cattle and dairy product utilization by smallholder farmers. Am.-Eurasian J. Sci. Res..

[54] Lonsdale C. (1989).

[55] Van Soest P., Robertson J. (1985).

[56] Berhanu A. (2014). Microbial profile of tella and the role of gesho (*Rhamnus prinoides*) as bittering and antimicrobial agent in traditional tella (Beer) production. Int. Food Res. J..

